# Critical Review on Physiological and Molecular Features during Bovine Mammary Gland Development: Recent Advances

**DOI:** 10.3390/cells11203325

**Published:** 2022-10-21

**Authors:** Shalini Jaswal, Manoj Kumar Jena, Vijay Anand, Avinash Jaswal, Sudhakar Kancharla, Prachetha Kolli, Gowtham Mandadapu, Sudarshan Kumar, Ashok Kumar Mohanty

**Affiliations:** 1 Proteomics and Structural Biology Lab, Animal Biotechnology Centre, National Dairy Research Institute, Karnal 132001, Haryana, India; 2 Department of Biotechnology, School of Bioengineering and Biosciences, Lovely Professional University, Phagwara 144411, Punjab, India; 3 Department of Veterinary Physiology and Biochemistry, Veterinary College and Research Institute (TANUVAS), Orathanadu 614625, Tamil Nadu, India; 4 National Referral Center, Dairy Microbiology Division, National Dairy Research Institute, Karnal 132001, Haryana, India; 5 Devansh Lab Werks, 234 Aquarius Drive, Homewood, AL 35209, USA; 6 Microgen Health Inc., 14225 Sullyfield Cir Suite E, Chantilly, VA 20151, USA; 7 ICAR-Indian Veterinary Research Institute, Mukteswar Branch 263138, Uttarakhand, India

**Keywords:** bovine, mammary gland development, mammary hierarchy, differentiation, lactation, involution

## Abstract

The mammary gland is a unique organ with the ability to undergo repeated cyclic changes throughout the life of mammals. Among domesticated livestock species, ruminants (cattle and buffalo) constitute a distinct class of livestock species that are known milk producers. Cattle and buffalo contribute to 51 and 13% of the total milk supply in the world, respectively. They also play an essential role in the development of the economy for farming communities by providing milk, meat, and draft power. The development of the ruminant mammary gland is highly dynamic and multiphase in nature. There are six developmental stages: embryonic, prepubertal, pubertal, pregnancy, lactation, and involution. There has been substantial advancement in our understanding of the development of the mammary gland in both mouse and human models. Until now, there has not been a thorough investigation into the molecular processes that underlie the various stages of cow udder development. The current review sheds light on the morphological and molecular changes that occur during various developmental phases in diverse species, with a particular focus on the cow udder. It aims to explain the physiological differences between cattle and non-ruminant mammalian species such as humans, mice, and monkeys. Understanding the developmental biology of the mammary gland in molecular detail, as well as species-specific variations, will facilitate the researchers working in this area in further studies on cellular proliferation, differentiation, apoptosis, organogenesis, and carcinogenesis. Additionally, in-depth knowledge of the mammary gland will promote its use as a model organ for research work and promote enhanced milk yield in livestock animals without affecting their health and welfare.

## 1. Introduction

The mammary gland, a unique organ in mammals, is a derivative of ventral skin [1,2]. The bovine mammary gland is composed of parenchymatous and stromal compartments. The parenchyma is a cellular compartment that contains two main cellular lineages. The major compartment is made up of inner luminal cells that surround a central lumen, while the minor compartment is made up of outer myoepithelial cells that are found at the base of the mammary epithelium, adjacent to the basement membrane (BM), and separate the mammary epithelium from the stroma. The luminal cells can be further divided into ductal (lining the lumen of ducts) and alveolar (milk-synthesizing cells) subtypes. The stromal compartment, unlike the parenchyma, is made up of a variety of cells (fibroblasts, mesenchymal cells, adipocytes, leukocytes, and blood cells) as well as extracellular matrix (ECM) (laminin, fibronectin, collagen, proteoglycan, etc.) [3,4].

**Figure 1:**
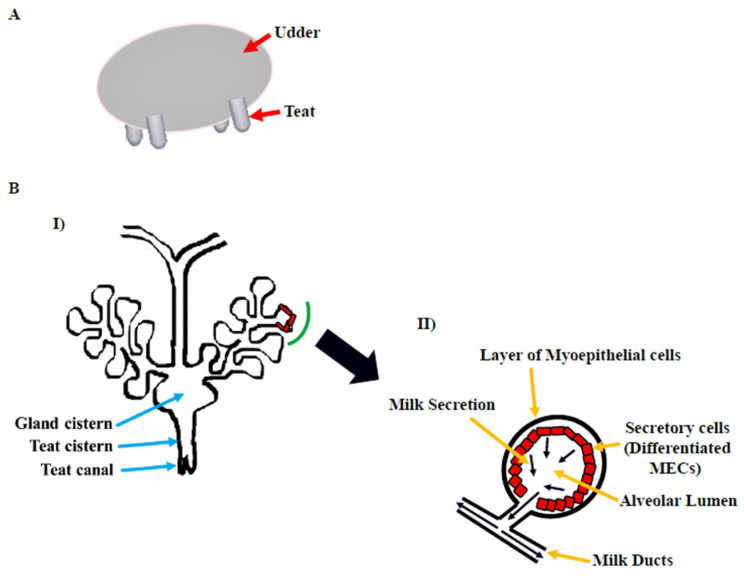
Bovine mammary gland. (**A**) Diagram illustrating the bovine udder. (**B**) I: Microscopic anatomy of bovine mammary gland consisting of milk ducts, alveoli, cistern (gland and teat), and teat canal. II: Secretory unit of mammary gland, i.e., alveolus containing milk-secreting mammary epithelial cells (MECs), covered by the myoepithelial cells which are in direct contact with extracellular matrix (ECM) [6].

The gross morphology of the mammary gland varies by species, but the microscopic structure is nearly identical. The morphology of the bovine mammary gland is more similar to that of the human breast in terms of the functional unit and stroma composition [5]. The bovine mammary gland, also known as the udder, is divided into two equal and distinct halves by a median suspensory ligament. Each half has two glands, each leading to a teat (Figure 1A). The teat consists of a cistern and canal and an aperture through which milk is discharged. The udder is attached to skeletal muscles, which support its large size [6]. The alveolus is the functional unit of the lactating mammary gland (Figure 1B). A single layer of cuboidal to columnar luminal mammary epithelial cells (MECs), which are primarily engaged in milk synthesis and secretion, line each alveolus. The myoepithelial cells, which have smooth muscle-like properties, surround these cells. These cells form a continuous barrier over epithelial cells in the ducts, preventing them from accessing the BM. In alveoli, the myoepithelial cells form a discontinuous layer so that the MECs are in direct contact with the underlying BM, which plays a crucial role in their differentiation [7]. The capillary network surrounds the alveoli, from which the layers of MECs take milk component precursors for lactose, protein, and milk fat synthesis. Suckling by pups causes the anterior lobe of the pituitary to release oxytocin hormone [8], which binds to oxytocin receptors in the mammary gland [9] and causes myoepithelial cells to contract in lactating mammary glands [10]. This compression aids in the ejection of milk from luminal epithelial cells into the alveolus and subsequently to the milk ducts and teat lumen [6,11].

The molecular alterations associated with each developmental stage of the cow mammary gland have been discussed in depth in this article. In addition, the regulatory molecules that control the shift from one stage of development to the next have been highlighted. Additionally, the important morphological changes in the development of the mammary gland have been thoroughly discussed. Altogether, the present review will help researchers compare the physiology of the cow udder to that of other animals, allowing them to better understand the differences in mammary gland biology.

## 2. Mammary Hierarchy

### 2.1. Mammary Stem Cells

**Figure 2:**
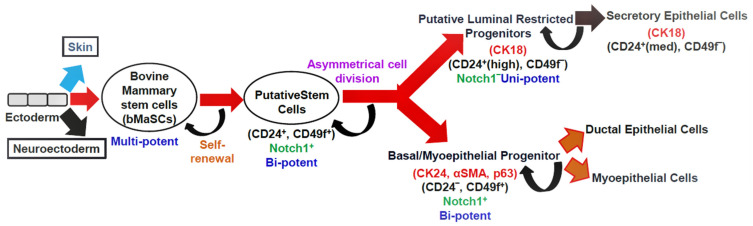
The hypothetical model depicting the mammary epithelial hierarchy in the bovine mammary gland. Cell surface markers: CD24, CD49f; lineage markers: CK18, CK14, αSMA, and p63; CK: cytokeratin, αSMA: smooth muscle alpha-actin, p63: tumor protein p63; med: medium expression.

Mammary gland homeostasis and regeneration are maintained by the controlled activity of stem cells [12,13]. Cluster of differentiation 24 (CD24) (heat-stable antigen) and CD49f (α6-integrin) are cell surface markers that distinguish mammary stem cells (MaSCs). These bovine MaSCs (bMaSCs) are multipotent and give rise to bipotent putative stem cells. The putative stem cells differentiate into luminal and basal progenitors. These progenitors differ in the type of cell surface and lineage markers they express (Figure 2). The luminal restricted progenitors are unipotent in nature and differentiate into secretory epithelial cells, whereas the basal progenitors are bipotent and can give rise to ductal epithelial cells and myoepithelial cells. The NOTCH signaling pathway in the putative stem cells and basal progenitors helps in self-renewal [14,15]. Both bovines and humans have a similar developmental hierarchy [16].

#### 2.1.1. Estrogen Receptor

In the mammary gland, 30–50% of MECs express estrogen receptor (ER) and progesterone receptor (PR) [17]. Two distinct genes on different chromosomes encode two different isoforms of ER, ERα and ERβ. In bovines, ERα is mostly expressed in luminal MECs, certain fat pad adipocytes, and fibroblast cells. In other animals, including humans, monkeys, and mice, its expression is restricted to luminal epithelial cells. ERβ, on the other hand, is mostly expressed in luminal epithelial, myoepithelial, stromal, and fibroblast cells, and its quantity in the bovine mammary gland is significantly lower than that in humans, monkeys, and mice [18].

17β-estradiol (E2) is an important regulator of mammary gland development that works through the ER [19]. In ERα^+^ and ERβ^+^ cells, E2 stimulates proliferation and apoptosis, respectively [20]. The non-pregnant heifer has a higher level of ERα expression, which decreases during lactation and involution [21,22]. ERβ expression, on the other hand, is stable throughout the stages of mammary gland development, except during the conclusion of udder feeding, when it is expressed considerably [21]. ER^+^ cells coexist with ER^−^ cells in close proximity. These cells release paracrine chemicals that regulate ER^−^ cell proliferation after being activated by E2 [17].

#### 2.1.2. Progesterone Receptor

Progesterone receptor (PR) is mostly expressed in epithelial, stromal, vascular, and fat cells in bovine [18]. Its expression is restricted to the luminal epithelial cells in other animals such as mice, monkeys, and humans. In the mammary gland, A and B isoforms of PR exist in a specific ratio that varies by species [18], with a 3:1 ratio in bovines [21] and mice [23]. Three different PR isoforms, namely A, B, and C, are expressed in the mammary gland of non-pregnant heifers; however, only the B isoform is expressed during lactation and involution [21].

## 3. Structural and Functional Development of the Bovine Mammary Gland

In terms of physical anatomy, location, and hormonal requirements, mammary gland development varies among species [24]. In ruminants, the mammary gland is positioned in the inguinal region; in pigs, rats, and mice, it is located in the torso region; and in elephants and primates, it is located in the thoracic region [2]. Unlike other organs that grow during embryogenesis, the mammary gland develops most of its characteristics after birth before fully maturing during pregnancy [25,26].

Ruminants, humans, and rodents have comparable mammary gland growth patterns [27,28]. Prepuberty, postpuberty, gestation, and lactation are the four stages of its development [29]. Several factors, including endocrine, autocrine, paracrine, intracellular factors, and extracellular matrix (ECM), regulate each developmental stage [26]. Following cycles of pregnancy, parturition, lactation, and involution, the female mammary gland experiences repeated rounds of apoptosis and growth. As a result, it is a fantastic model for researching stem/progenitor cells that can expand and replenish themselves repeatedly [30].

### 3.1. Morphological and Molecular Events during Different Stages of Bovine Mammary Gland Development and Their Regulation

#### 3.1.1. Embryogenesis

**Figure 3:**
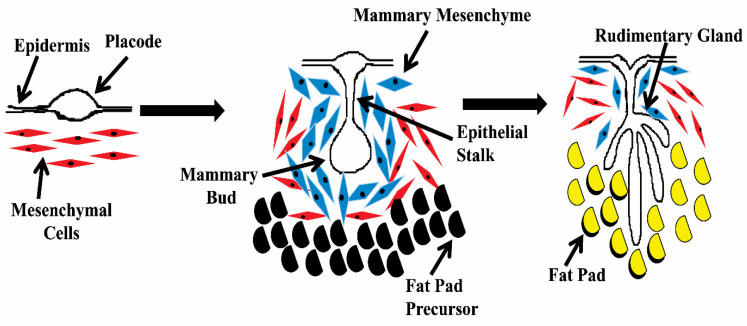
Schematic diagram illustrating the embryonic development of the bovine mammary gland. The mammary placode expands into the mesenchyme and the rudimentary structure surrounded by the fat pad [35].

The formation of two symmetric milk lines from the overlaying ectoderm initiates the development of the mammary gland in a bovine when the fetus is between 1.4 and 1.7 cm long. Milk lines occur between the fore and hind limbs at 10, 30, and 34 days of gestation in mice, bovines, and buffaloes, respectively. They disappear after a short time, around 35 to 42 days after conception, when the embryo reaches a length of 2.0 to 2.1 cm. Subsequently, placodes, which are minute lens-shaped thickenings, grow along the milk line. Placodes are made up of multilayered columnar cells that arise as a result of cell migration inside the milk line. The number of pairs varies by species, with one pair in humans and five pairs in mice [31]. Furthermore, mesenchymal cells around the placode condense and form mesenchyme. Each mammary placode invades the underlying mesenchyme and increases in size to form the bud. In the bovine embryo, buds are formed when the fetus is 5–10 cm in length, around 49–56 days post-conception. After that, the rapid proliferation of epidermal cells gives rise to a primary sprout at the proximal end of the bud around 62 days post-gestation in buffalo. Furthermore, proliferation leads to the outgrowth of the primary sprout to the teat. Subsequently, its luminization gives rise to the primary duct. The terminal end of the primary sprout leads to the formation of secondary sprouts, which subsequently form milk ducts. However, the primary sprouts continue to penetrate the developing dermis without creating secondary sprouts in many eutherians, including cattle and mice. The formation of a rudimentary mammary gland structure in the growing fat pad is the consequence of repeated sprouting and branching of buds (Figure 3). A major duct with 10–15 tiny branches of ductal trees buried in the fat pad makes up this structure. The rudimentary ductal tree grows isometrically until puberty, when it provides the foundation for the mammary gland’s subsequent development [31,32]. In most animals, the development of the embryonic mammary gland is nearly identical in males and females. On the other hand, female cattle have elongated and ovoid mammary buds that grow earlier than those of males [33]. In male cattle, when the androgen hormone testosterone interacts with receptors on myoepithelial cells during embryonic development, sexual dimorphism is achieved. Myoepithelial cells undergo apoptosis and the mammary mesenchyme condenses due to this interaction. Later on, the volume of breast epithelium is reduced, and the mammary rudiment–epidermis link is ruptured [34]. In humans, however, sexual dimorphism is delayed and occurs by a distinct process [35].

The development of the mouse’s embryonic mammary gland is not dependent on hormones but on reciprocal signaling between the mammary epithelium and the mesenchyme [36]. The milk line is partially established via the non-canonical wingless-related integration site (WNT) pathway, specifically WNT10b in the mesenchyme and epithelium [37]. The fibroblast growth factor (FGF) regulates the activity of WNT10b. T box (Tbx) proteins are induced and maintained by FGF receptor 1 (FGFR1). These proteins regulate the dorsoventral location of placodes and are expressed in the mesenchyme beneath the milk line. FGF10b stimulates the formation of placodes 1, 2, 3, and 5, which are only expressed in the somites beneath the mammary line in mice. Furthermore, parathyroid hormone-like hormone (PTHLH) and bone morphogenic protein 4 (BMP4) promote bud sprouting, ductal expansion, and the development of nipples [2]. PTHLH also promotes the production of muscle segment homeobox 2 (Msx-2), a transcription factor that limits the formation of hair follicles on teats [35].

#### 3.1.2. Prepubertal Mammary Gland Development

In heifers, early pubertal growth of the mammary gland occurs in two phases, depending on how fast it grows compared to the rest of the body. When the mammary gland (only non-epithelial tissue) grows at the same rate as the rest of the body, the increase is isometric. Isometric growth results in the development of the fat pad and circulatory system in the mammary gland. At the age of 2–3 months, a heifer’s mammary gland begins allometric growth, which lasts for up to 9 months. At this point, the rate of growth of the mammary gland is 3 to 5 times quicker than the rest of the body, resulting in substantial mammary gland proliferation, development of the ductal network, and increased size of the fat pad [38]. This is accompanied by a significant increase in prolactin levels at the age of three months, and these levels approach a plateau at nine months [39]. During allometric growth, high planes of nutrition have a negative impact on udder development as compared to heifers fed a restricted diet. In prepubertal heifers, this results in the development of excessive fat content and decreases milk-producing tissue in the mammary gland, lowering milk output [40]. A heifer’s gland weighs about 2–3 kg after allometric growth, and the parenchymatous tissue is normally made up of 40–50% connective tissue, 30–40% adipocytes, and 10–20% epithelial cells [38].

The growth of the mammary gland during prepuberty is controlled by the combined actions of GH, estrogen, and IGF-1. Estrogen induces rapid proliferation of ducts expressing ERα. The proliferative action of estrogen is mediated by the insulin-like growth factor-1 (IGF-1) protein (IGF-1). The significance of estrogen was further elucidated by ovariectomy which reduces proliferation in MECs and impairs mammary gland development [18,41].

#### 3.1.3. Pubertal Mammary Gland Development

The majority of mammary gland development occurs in the postnatal period, when there is a rapid growth of ducts. Ductal growth requires a fat pad; however, in heifers, the small size of the fat pad restricts extensive ductal growth. Heifers enter puberty at the age of 9–10 months when udder growth returns into the isometric phase [38]. Subsequently, the rapid growth of ducts from the teat gives rise to a tree-like network in the surrounding adipose-rich stroma [35]. The primary ducts are surrounded by alveolar epithelial cells, which are further surrounded by a thick layer of the dense stroma composed of adipocytes, fibroblasts, ECM, and capillaries. The primary ducts have a large lumen and serve as a reservoir for milk during lactation [38]. Terminal end buds (TEBs) bifurcate into secondary ducts with independent TEBs during primary duct growth [42,43]. Secondary duct branching develops and continues until the fat pad is densely filled with the ductal system, leaving some room to be filled with ducts during pregnancy [5,44]. Puberty results in the formation of bilayered tiny ductal trees in the adipose tissue, which are made up of luminal and myoepithelial cells [35,45].

**Figure 4:**
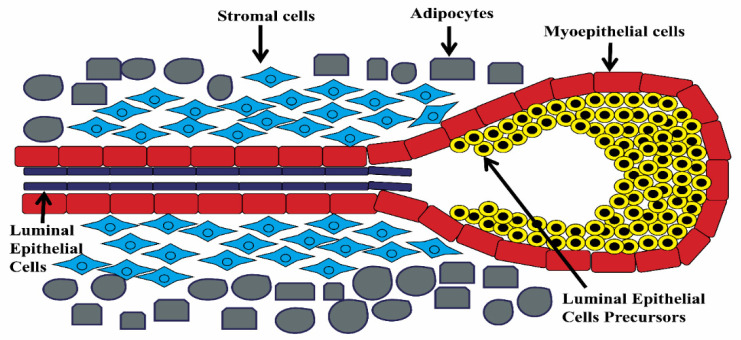
Schematic diagram representing the terminal end bud (TEB).

In addition to endocrine factors, the paracrine interactions between the growing ducts and the surrounding stroma influence puberty growth. In the presence of the ovarian hormone estrogen, pubertal mammary gland growth begins. The expression of transmembrane amphiregulin (AREG) is induced when it binds to the ER. This results in the production of epidermal growth factor (EGF), which then drives cell proliferation and the formation of TEBs at the duct tips. TEBs have two types of histologically distinct and specialized epithelial cells: body and cap. A single layer of cap cells surrounds the multilayered MECs at the distal end of TEBs. These cap cells are the progenitors for both luminal and basal epithelial cells (Figure 4) [42]. By paracrine action, activated ER^+^ cells produce growth factors (GFs), which aid in the proliferation of ER- cells [46]. NOTCH signaling is also involved in cell–cell interactions, cell proliferation, differentiation, and stem cell maintenance through NOTCH receptor paralogues 1 to 4. In humans, mice, and cattle, this plays a vital function in regulating the growth of the mammary gland during puberty [47].

Heifer growth is also influenced by the amount of growth hormone (GH) in the blood. A heifer with a high GH level grows more quickly. GH promotes the growth of parenchymatous tissue but inhibits the growth of adipose tissue [48]. In stromal fibroblasts, GH binds to the GHR and stimulates the synthesis of IGF-1 [35]. IGF-1 is an endocrine and paracrine hormone that mediates the activity of GH [49]. It communicates with epithelial cells via IGFR1 [28]. MEC proliferation in TEBs is regulated by GH, IGF-1, and estrogen for ductal expansion and morphogenesis [35,38]. In other species, such as rats and monkeys, prolactin rather than GH plays an important role in mammary gland growth [48].

Other factors that have been shown to play a role in branching morphogenesis during puberty include netrin 1 (NTN1), slit2 (SLIT2), reelin (RELN), milk fat globule-EGF factor 8 protein (MFGE8), and matrix metalloproteinases (MMPs) [35]. In the branching morphogenesis of the mammary gland, canonical WNT signaling plays a key role. Loss of WNT-5a in TEBs activates β-catenin, which, once translocated into the nucleus, causes the production of cell cycle progression markers and hence promotes MEC proliferation [50]. Pubertal growth includes the construction of the vascular and lymphatic networks in the mammary gland during puberty, in addition to the development of ducts. The myoepithelial cells produce vascular endothelial growth factors (VEGFs) C and D, which regulate the process [30]. The endogenous production of transforming growth factor-beta (TGF-β) inhibits further enlargement of the fat pad once it has been filled with the mammary ductal system [51].

#### 3.1.4. Pregnant Mammary Gland Development

During pregnancy, extensive breast tissue remodeling occurs, and the mammary gland develops in three stages: early, mid, and late. During the early stages of pregnancy, the MECs in the mammary gland proliferate rapidly, resulting in the formation of alveolar structures at the ducts’ terminal ends. The connection between alveoli and primary milk-collecting ducts is developed during early pregnancy by lateral branching in the vast ductal system [52]. MMP-3 is responsible for the 2 and 3° lateral branching [53]. The activated MMPs trigger a proteolytic cascade in MECs that causes EMT by lowering E-cadherin and β-catenin expression and increasing vimentin expression. This aids MEC movement, which is required for branching morphogenesis [54]. The ECM remodeling resulting from increased MMP14 expression in TEBs aids the branching process [55]. As the alveolar epithelial cells enter the differentiation phase, the proliferation of the cells decreases dramatically by mid-pregnancy. MECs undergo terminal differentiation and gain the ability to produce milk proteins throughout late pregnancy [56]. The extensive proliferation of MECs is followed by their terminal differentiation during the late stages of pregnancy. To understand the protein machinery governing the differentiation in buffalo mammary epithelial cells (BuMECs), a differential proteome study was performed by Jaswal et al. (2021) [57]. The tandem mass tag (TMT)-based differential proteome analysis of hormone-treated (prolactin, 8 µg/mL; bovine insulin, 1 µg/mL; and hydrocortisone, 1 µg/mL) BuMECs at different time points of 3, 6, 12, and 15 days identified a total of 4934 proteins; of them, 681 were differentially regulated. The study reported a significantly high level of expression of a few uncharacterized proteins, including KH domain-containing RNA-binding, signal transduction-associated protein 1 (KHDRBS1) (FC 88.15), ATP-binding cassette sub-family A member 13 (ABCA13) (FC 84.57), and LOC519132 (FC 42.29), which can be considered as the potential biomarkers for the differentiation but need further study. In addition, the preliminary study suggested the role of involucrin in assisting the differentiation of BuMECs. The differential proteome dataset can be used to understand the species-specific variations among lactating animals such as goats, cows, and humans.

In bovines, estrogen and progesterone are important hormones for the development of the pregnant udder [21]. The estrogen level in the blood plasma of dairy cows rises gradually from 110–120 days of pregnancy to 40 pg/mL during late pregnancy (230–250 days) [58] and remains stable until 16–18 days before delivery [59]. The buffalo follows a similar expression pattern, with plasma levels of estradiol increasing within a day following insemination and fluctuating between 8.29 and 13 pg/mL throughout the first trimester. During the second trimester, estradiol concentration rises to a higher level than in the first trimester, reaching its peak a day before parturition. Following that, its level drops dramatically the day after parturition and remains stable for the next 6 days [60]. The plasma level of estrogen in the cow is quite low in comparison to other species such as humans (100 pg/mL) and monkeys (300–400 pg/mL) [58,61]. From 90 to 150 days of pregnancy, the plasma content of progesterone in dairy cows increases slightly and is maintained 7 to 14 days prepartum. Following that, its level gradually drops, followed by a sharp drop two days before parturition [62]. On the other hand, by day 13 of insemination, the level of progesterone in buffalo reaches its maximum and is maintained throughout the first, second, and third trimesters of pregnancy, followed by a quick fall at parturition and a large increase 16 days postpartum [60]. Overall, the plasma progesterone level is high and stable throughout pregnancy, but the estrogen level is low in the first trimester and then rises in the second half [63].

In 2012, Huang et al. [64] used mass spectrometry (MS) to examine the nuclear proteome of β-estradiol-treated dairy cow MECs (DCMECs) and identified seven differentially expressed proteins (DEPs), including glycyl-tRNA synthetase and nuclear GTP-binding proteins. The study revealed that estrogen impacts the expression of phosphorylated nuclear proteins, which could help in better understanding how estrogen affects milk protein synthesis [65]. Estrogen works through both paracrine and autocrine processes, resulting in ductal and alveolar expansion in the first half of pregnancy [66,67] and lobuloalveolar growth in the second half [68]. Through the receptor activator of nuclear factor kappa-β ligand (RANKL) signaling system, progesterone aids in alveolar development. The binding of RANKL to its receptor RANK stimulates the production of cell cycle-related genes such cyclin D1 [69,70]. Interestingly, alveoli contain both PR^+^ and PR^−^ cells, with PR^−^ cells requiring the paracrine pathway to proliferate. The interaction of progesterone with PR promotes PR^+^ cells, releasing paracrine factors such as WNT4 and nuclear factor (NF)-B ligand (RANKL). The growth of PR^-^ cells is aided by these stimuli [71]. In addition to its role in lobuloalveogenesis, progesterone inhibits the transcription of milk protein genes such as α-lactalbumin (LALBA) [71] and β-casein (CSN2) during pregnancy [72]. Progesterone prevents cortisol from attaching to an intracellular receptor, preventing cortisol and prolactin from working together to initiate milk release. After delivery, progesterone levels drop, which permits cortisol to bind and start lactation [65].

MECs undergo a variety of alterations at the molecular and cellular levels, as well as in their morphology, during terminal differentiation in the late pregnancy. Because of the failure of cytokinesis under the action of aurka kinase A, these cells are binucleated with 4n DNA content. About 17% of alveolar MECs in mice are binucleated at 18.5 days of pregnancy, but this percentage rises to 50% on the second day of lactation, when the mammary gland enters the secretory phase. During breastfeeding, roughly 30% of bovine alveolar MECs are binucleate, whereas about 40% of human, seal, and wallaby MECs are binucleate [73]. Increased mitochondrial size and accumulation of cellular organelles such as the endoplasmic reticulum, Golgi apparatus, and secretory vesicles are among the cellular alterations. The nuclei of differentiated MECs translocate to the basal surface as a result of this. Many microvilli were seen on the apical surface of terminally differentiated MECs, providing a larger surface area for effective milk synthesis and secretion [74,75].

Only prolactin signaling has been extensively studied for its function in MEC differentiation and milk protein synthesis [76,77,78]. Lactotroph cells in the pituitary gland secrete prolactin, a 23 kDa peptide hormone [79]. Its expression in rats begins in the middle to late stages of pregnancy, continues through nursing, and then drops during the early stages of involution [80]. The plasma level of prolactin in cattle, on the other hand, is 9 ng/mL during gestation and rises to 76 ng/mL by 270 days. It reaches its peak level, commonly known as prolactin surge, 5 days before delivery and then steadily falls by 48 h after delivery. Its concentration varies by species, ranging from 25 ng/mL seven days before delivery in sheep to >200 ng/mL by 21 weeks in goats, and it is maintained during parturition unlike in cattle [81]. However, a digital gene expression (DGE) examination of bovine mammary tissue at three time intervals during mammary gland development (about day 35 before parturition, day 7 before parturition, and day 3 after parturition) revealed that Janus kinase 2 (JAK2) and signal transducer and activator of transcription 5A (STAT5) have a small function in udder feeding [82]. The expression of milk protein genes is temporal, with WDNM1 and CSN2 levels being higher in early pregnancy and whey acidic protein (WAP) and α-lactalbumin (LALBA) levels being higher in late pregnancy. Prolactin is one of the major molecules reported to regulate the differentiation of MECs in many lactating species. To uncover its mechanism of action, 2-DE proteomic analysis of nuclear phosphoproteins isolated from the dairy cow MECs (DCMECs) treated with prolactin was performed. The study reported seven significant DEPs (fold change ≥ 2), with a potential role in the lactation process. A few of these proteins were glycyl tRNA synthetase (GARS), serpin family H member 1 (SERPINH1), thioredoxin-dependent peroxide reductase, mitochondrial (PRDX3), actin-related protein 1A (ACTR1A), and annexin A2 (ANNEXINA2) [83].

Milk synthesis and secretion are controlled by a number of factors. Prolactin is the major hormone regulating the processes of milk protein synthesis, initiation of milk secretion (lactogenesis), and maintenance of milk secretion (galactopoiesis). In addition, GH can also regulate lactogenesis and galactopoiesis in the absence of prolactin hormone in ruminants, rats, and humans [84,85]. An MS study of GH-treated bovine mammary alveolar cell-T (MAC-T) cells revealed the key role of GH in their differentiation [86]. A few other factors, including amino acid and glucose transporters as well as insulin and mTOR signaling, also play a key role in the regulation of milk protein synthesis in the bovine mammary gland [87,88].

Studying mammary gland biology using in vivo tissue is constrained by the associated ethical issues in large animals. So, a large number of studies have been performed using in vitro models to understand the mechanism of differentiation in MECs. A few of them, including human MEC line MCF-10 [89], mouse MEC lines COMMA1D and HC11 (derivative clone of COMMA1D cell line) [90], buffalo MEC line BuMEC [91], bovine mammary alveolar cell-T (MAC-T) line [92], and yak MEC line (YMEC) [93], are available for use in studying the mammary gland biology. The deep proteome analysis of actively proliferating BuMECs using subcellular protein fractionation complexed with peptide fractionation and high-resolution MS identified a total of 12,609 non-redundant proteins. This study reported the presence of a few new proteins for the first time in BuMECs, including actin-binding LIM protein (abLIM), secreted phosphoprotein 1 (SPP1), osteopontin (OPN), furry homolog (FRY), FRY-like transcriptional coactivator (FRYL), UNC variants 79 and 80, sodium leak channel non-selective protein (NALCN), and aquaporin 6 (AQP6), which may have a crucial role in the mammary gland development. In addition, the presence of germ line-specific proteins such as testis-specific protein Y-linked 2 (TSPY2), a disintegrin and metalloprotease domain-containing protein (ADAM), ATP-dependent RNA helicase VASA (VASA), and JY-1 suggested their possible role in the active proliferation of BuMECs. The huge dataset can be used for proteome-based genome annotations, and for parallel comparative studies in other species [94]. The growth of dome-like structures and the synthesis of CSN2 in MECs is regarded as indicative of functional differentiation in an in vitro system [95]. Dome structures are three-dimensional structures generated on a confluent monolayer of MECs in culture, with a single layer of cells above the monolayer and a fluid-filled lumen. The ionic insulation of the inner fluid from the bulk culture media is maintained by the impermeable tight connections between the cells composing the dome. Dome cells are polarized and have closed tight connections, whereas monolayer cells have open tight junctions [96,97]. Domes range in size from 75 μm to 1 mm in diameter, with polarized cells being 1.5 to 2 times bigger than monolayer cells [98,99]. Lactogenic hormones such as prolactin and insulin, as well as chemicals such as dimethyl sulfoxide (DMSO), have an effect on dome formation [100]. Parturition occurs after the pregnancy stage and is the transition period between late pregnancy and lactation. Parturition, often known as the secretory phase, entails a large amount of milk secretion. During parturition, terminally differentiated MECs synthesize and secrete a high number of milk proteins [101].

#### 3.1.5. Lactating Mammary Gland Development

The lactating mammary gland is made up of 40–50% epithelial cells, 40% connective tissue, and 15–20% lumen [38]. Lactogenesis is the process of the secretory MECs synthesizing and secreting milk protein during the transition from pregnancy to lactation [102]. Lactogenesis is divided into two stages: lactogenesis I and lactogenesis II. The MECs acquire limited synthesis and secretion capabilities during lactogenesis I, also known as secretory differentiation [103]. The expression of milk proteins such as CSN and LALBA, the secretion of pre-colostrum, and a poor contact between MECs and ECM characterize this phase [82]. It occurs in dairy cows during late pregnancy [82], in humans during mid-pregnancy [104], and in rats ten days after pregnancy [105]. The ability of MECs to secrete milk protein is limited by high progesterone levels in the blood [106].

Lactogenesis II, also known as secretory activation, begins around parturition in lactating animals, such as cows, with a quick fall in plasma progesterone levels, but it occurs four days later in humans, as evidenced by a rise in milk supply from 100 mL on day 1 to 500–750 mL/day [104]. The highly impermeable tight connections between the MECs in the alveoli characterize this phase, which is required for the directed secretion of colostrum (immunoglobulins, sodium, and chloride ions) followed by milk (lactose, glucose, and potassium ions) into the lumen [107]. MEC–ECM contact is restored [82,102], which aids in the uptake of nutrients from the circulation by MECs for the production of milk components [108].

**Figure 5:**
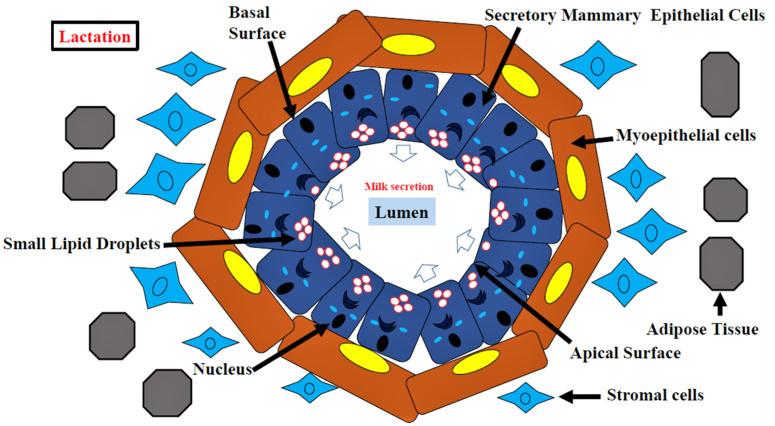
Schematic diagram illustrating the MECs in the lactating mammary gland. White arrows depict the direction of milk secretion from the apical surface of secretory epithelial cells.

The MECs go through many alterations at the molecular level during lactation. The elevated expression of genes involved with transport activities, such as glucose transporter (GLUT1), required for glucose uptake by MECs for lactose synthesis, was found in Holstein cow mammary tissue on day 5 prepartum and day 10 postpartum [109]. E74-like factor 5 (ELF5) acts as the major regulator of milk protein synthesis in the cow mammary gland during lactation [86], whereas sterol regulatory element-binding protein (SREBP) tightly regulates lipid synthesis [82]. During nursing, large lipid droplets in differentiated MECs break down into small droplets and collect towards the apical surface, in contrast to alveolar MECs during late pregnancy [56,73] (Figure 5). The milk protein profile at different periods of lactation, including early, peak, and late-stage variations, works as a signal of mammary gland intracellular modifications. The protein profile of bovine MECs differed between these stages, influencing metabolic processes, binding, and catalytic activities in udder cells, according to MS analyses. AKT, phosphatidylinositol-3 kinase (PI3K), and p38 mitogen-activated protein kinase (p38 MAPK) are only a few of the signaling pathways involved in the lactation process [110].

In a recent study [111] on BuMECs, it was revealed that the enzyme elongase (responsible for long-chain fatty acid synthesis) promotes lipid synthesis during lactation by synthesizing long-chain fatty acids and also regulating the expression levels of other related genes for milk fat synthesis during lactation. A comparative proteome study of exosome-derived proteins (exosomes isolated from cultured bovine mammary epithelial cells (BMECs) and milk) revealed the involvement of exosome proteins in signaling pathways regulating the lactation process [112]. An LC-MS study of BMEC-derived proteins identified 638 proteins, and a comparative proteome study with milk exosome-derived proteins revealed 77 proteins in common in both sources. This study showed that exosomes might play role in milk synthesis during lactation. The protein MFGE8 is a glycoprotein related to the milk fat globule membrane; its overexpression is indicative of high milk yield in animals. A study on the downregulation of MFGE8 in cultured buffalo MECs and further proteome analysis revealed the downstream targets of MFGE8 involved in the regulation of MEC cell physiology [113]. This study showed that MFGE8 knockdown disturbs many intracellular signaling pathways, leading to the cessation of cell growth. Bioinformatics analysis revealed that MFGE8 is activated by chemokine fractalkine (CX3CL1), tumor protein 63 (TP63), and colony-stimulating factor 2 (CSF2), which leads to the activation of suppressor of cytokine signaling 3 (SOCS3) and chemokine (C-C motif) ligand 2 (CCL2) for the regulation of cell proliferation. Furthermore, MFGE8 knockdown activated three independent signaling pathways, namely the ZP4/JAK STAT5, dedicator of cytokinesis 1 (DOCK1)/STAT3, and phosphatidylinositol (3,4,5)-triphosphate (PIP3)/AKT/mammalian target of rapamycin (mTOR) pathways.

In lactating animals, metabolism is highly activated under the influence of hormones and suckling [114]. This is in accordance with the study conducted by Jena et al. (2015) [115], which identified 21 upregulated proteins in the lactating as compared to the heifer’s mammary gland tissue using MS and reported that the majority of them were involved in the metabolic processes. In another study, one-dimensional sodium dodecyl-sulfate polyacrylamide gel electrophoresis (1-DE) coupled with LC-MS/MS proteomic analysis of microsomes from lactating bovine mammary tissue identified a total of 703 proteins, of which more than 50 proteins were associated with metabolism, cellular uptake, and secretion of lipids [116]. The above results are also supported by those of Finucane et al. (2008) [109], who performed a microarray analysis of the bovine mammary gland from late pregnancy to lactation on day 5 before parturition and day 10 after parturition. Their data revealed that the upregulated genes were associated with carbohydrate metabolism (acetyl-coenzyme A synthetases, 6-phosphofructo-2-kinase, etc.) and the transport activity of amino acids and lipids, whereas the downregulated genes were involved in the cell cycle and cell proliferation (cell division cycle associated proteins, cyclins, etc.) and protein and RNA degradation (proteasome, RNA binding motif protein, etc.). In another study, microarray analysis of the bovine liver and mammary gland during lactation was performed to understand the difference in metabolic regulation and to determine the profile of metabolic-related genes. The results demonstrated that the differentially expressed genes (DEGs) upregulated in the liver were involved in carbohydrate, lipid, and amino acid metabolism. Likewise, the DEGs upregulated in the mammary tissue were found to be involved in amino acid and sugar transporters and MAPK, WNT, and JAK-STAT signaling pathways. This study simplifies the understanding of the molecular basis of metabolic adaptation of the liver and mammary gland during lactation in bovine species [117]. The differential proteome analysis of the mammary gland suggested the higher upregulation of metabolic processes such as glycolysis, the tricarboxylic acid (TCA) cycle, and oxidative carboxylation during the lactation period [118]. This is in accordance with the study by Bionaz et al. (2012) [119], which states that the energy and protein requirement increases 5 times from late pregnancy to lactation in dairy cows. Leptin is a protein hormone secreted by the white adipose tissue, and it regulates metabolism during lactation. Its expression is regulated by the prolactin in the bovine mammary gland [120]. Similarly, rodent lactating MECs are metabolically highly active in milk synthesis, which is supported by insulin [106]. The increased blood flow during lactation serves as the source of nutrients required for the synthesis of milk components. In a cow, 400 L of blood flow through the mammary gland for the synthesis of 1 L of milk [27].

The MS approach has helped in understanding the protein profiles among different stages of mammary gland development. MS analysis of mammary gland tissues from lactating and involutory stages identified a total of 60 DEPs; of them, 57 were highly upregulated during lactation and 3 were downregulated. The majority of the proteins were associated with metabolic activities, which might be essential for the synthesis and secretion of milk proteins. The study resulted in the identification of five novel proteins that may have a crucial role in the lactation process [118]. Milk proteome studies have revealed the presence of many newly discovered proteins through the MS approach. The protein profile in milk reflects the mammary gland’s health along with the richness of the milk that is produced by the animals. The comprehensive milk proteome study by Rahman et al. (2021) [121] of the small extracellular vesicles of late-stage lactating cows observed many newly identified proteins (429) along with the identification of a total of 2225 proteins in total proteome analysis. Thorough in silico analysis of the newly discovered proteins showed their involvement in physiological processes during milk production and immune response mechanisms.

Lactation in bovines is a lengthy process that lasts more than 300 days [122]. During this stage, the milk yield profile follows a typical relationship with progress in time. Peak lactation in dairy cows is followed by a rapid decline in milk production, which coincides with a decrease in estrogen levels [29]. The role of STAT5 in milk protein synthesis was confirmed by Lu et al. in 2012 [123]. The phosphoproteomics of dairy cow MECs (DCMECs) treated with 1.2 mM L-lysine (L-Lys) identified six upregulated proteins in L-Lys-treated DCMECs. Silencing of MAPK1 using siRNA showed that it functions through the STAT5 and mTOR pathways. In a similar study by Lu et al. (2012) [124], the role of L-methionine in milk protein synthesis in dairy cow MECs (DCMECs) was studied. 2-DE/MS-based phosphoproteomics analysis revealed the upregulation GARS, septin-6, staphylococcal nuclease domain-containing protein 1 (SND1), twinfilin-1, and eukaryotic elongation factor 1-beta (eEF1B). However, detailed study on the regulation of milk protein synthesis at the transcript and translation levels is still required.

To better understand lactation biology, the dynamic changes in the expression of proteins were studied in the MECs isolated from the milk during three stages of lactation, i.e., immediate early, peak, and late. The study identified 41 DEPs among the three stages of lactation, which mainly showed enrichment in binding and catalytic activities and play essential roles in cell–cell and cell–ECM interactions and in metabolic processes. The proteins identified during the late lactation stage were reported to be involved in the nuclear factor kappa beta (NF-κβ) and JNK-MAPK pathway, which triggers the cellular stress and apoptosis of MECs. In addition, a study was conducted to analyze the changes in the expression of proteins among high- and low-yielding cows. The proteins highly abundant in the high-yielding cows were found to be associated with the AKT, PI3K, and p38/MAPK signaling pathways, which act through insulin hormone signaling [110]. In dairy cows, peak lactation is followed by an accelerated decrease in milk production, which coincides with the decrease in estrogen level. Hence, a high level of E2 negatively regulates lactation [29]. In a similar study, Zheng et al. (2017) [125] compared the proteome profile of the dairy cow mammary tissue during two lactation stages, i.e., peak and late. The study identified a total of 3753 proteins, of which 179 were differentially expressed. The downregulated proteins during the late lactation stage were found to be involved in the processes related to localization, lipid metabolism, and transportation. The processes such as apoptosis, and immunity were highly enriched during the late lactation period. Based on an analysis of quantitative trait loci and a genome-wide association study, 95 proteins were found to regulate the milking performance. The results suggested the occurrence of dynamic changes in the expression of proteins between the two stages of lactation. The LC-MS analysis of MECs isolated from milk using immunomagnetic beads identified 431 and 134 proteins by 1D and 2DE approaches, respectively. The association of these proteins with 28 signaling pathways may have a significant role in the lactation process of dairy cows [126]. The study by Silva et al. (2022) [127] established a delicate relationship between the nutrient intake and synthesis of milk components in bovine milk. This study involved bovine mammary epithelial cells and mammary tissue slices and found that decreased leucine intake activates the glucose uptake mechanism by glucose transporters (GLUT1), without affecting the lactose synthesis rates. However, a deficiency of isoleucine and valine along with leucine affected the lactose synthesis rate without hindering the glucose uptake.

Little information is available on the expression profile and role of plasma proteins in the lactating mammary gland. In one study, total protein profiling of plasma membrane (PM) fractions of the mammary gland in lactating cows was performed using 1D-LC-MS/MS. The identified proteins were found to be involved in transport, binding, and catalysis and included S100 calcium-binding proteins, annexins, integrins, and heat shock functions. This was the first study providing a reference for the PM proteins of the mammary gland in lactating cows [128]. Interestingly, the proteomic approach was used to identify the potential biomarkers in bovine milk that can be further used to study the animal’s health status. A total of 13 proteins were differentially expressed among the high-resistance and low-resistance animals. Lactoferrin was one of these potential biomarkers and was expressed at a higher level in the low-disease-resistance animals [129]. It is known that vacuolar protein sorting-associated protein 28 homolog (VPS28) regulates the synthesis of milk fat during lactation; however, its molecular mechanism of action is not clear. To study its mechanism of action, the VPS28 gene was knocked down in bovine primary MECs, and the resulting cells were analyzed using the isobaric tag for relative and absolute quantitation (iTRAQ). The study identified and quantified a total of 2773 proteins. Of these proteins, a total of 92 proteins were upregulated and 203 proteins were downregulated in the knocked-down cells as compared to the normal cells. Further, bioinformatics analysis suggested the involvement of these proteins in various biological functions such as proteolysis, metabolic reactions, and phosphorylation. In addition, the knockdown of this gene altered the morphology of BMECs, an affect the process of proteolysis. Altogether, the results of this study suggested that VPS28 functions in milk fat synthesis through the process of ubiquitination. This study will help to understand its role in detail [130]. The 2-DE and matrix-assisted laser desorption ionization (MALDI)-based proteome profiling of lactating bovine mammary gland tissue identified a total of 215 unique proteins. Of these proteins, 15 proteins with catalytic activities were identified as having a role in the metabolism of glucose to fatty acids (11 proteins), in the pentose phosphate pathway (2 proteins), or in the synthesis of lactose from glucose (2 proteins).

#### 3.1.6. Involutory Mammary Gland Development

The transformation of a milk-producing mammary gland into a non-functional pre-pregnant state is known as involution. During this period of development, the mammary gland experiences the most extensive tissue repair and histological changes. By day 2 of involution in bovine MECs, the formation of massive intracellular vacuoles as a result of coalescence among fat droplets and protein-rich secretory vesicles displaces the organelles towards the basal surface around the nucleus, and the organelles thereafter vanish within 1 to 2 weeks. The MECs are metabolically active, but due to a decrease in the number of organelles, such as the rough endoplasmic reticulum, Golgi apparatus, and mitochondria, they are unable to produce milk components. By day 21 to day 30 of involution, the alveolar structures collapse, and the increased inter-alveolar space is filled with fibrous connective tissue containing a few cells such as plasma cells, fibroblasts, phagocytes, and lymphocytes [74].

The mammary gland’s involution is a multistep process that occurs in two distinct physiological phases. The terminally developed epithelial cells undergo early apoptosis and the loss of tight junctions (TJs) between them in the first phase [131]. The stimulation of leaky TJs results in an increase in plasma electrolytes (Na^+^ and Cl^−^) as well as a large number of leukocytes in the mammary gland [132,133]. The degradation of CSN2 protein is catalyzed by the conversion of plasminogen to plasmin [132,134]. The peptide produced as a result of proteolysis also blocks potassium channels on the apical membrane of MECs, lowering milk production in cows and goats [135]. Tissue inhibitors of metalloproteinases (TIMPs) keep the matrix metalloproteinases (MMPs) inactive [131]. It has been proposed that injecting casein hydrolysate into the mammary glands of goats and cows causes milk stasis by triggering a series of processes. Involution in the bovine mammary gland is less widespread and occurs at a slower rate than that in the rodent mammary gland, as evidenced by a lower level of insulin-like growth factor-binding protein (IGFBP-5) expression and enhanced IGF1-AKT signaling [136,137]. The binding of IGFBP-5 to IGF-1 inhibits IGF-1 activity and promotes MEC apoptosis [138]. In mice, this phase of involution lasts 48 h and can be reversed if pups are permitted to suckle [131], whereas in bovines, the MECs are in a quiescent condition until 192 h after non-milking and can revert to milk synthesis once prompted by suckling [137]. When a nursing mother stops suckling, her hormones change, affecting the delivery of soluble survival growth factors such as IGF-1 [139].

**Figure 6:**
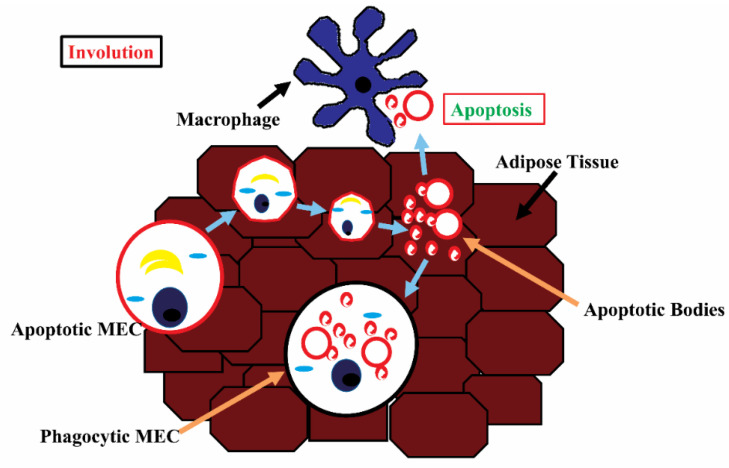
Schematic diagram illustrating the involutory bovine mammary gland containing macrophage and apoptotic and phagocytic MECs.

During the second phase, MMPs such as MMP3, plasmin, cathepsin B, and serine proteases, as well as apoptosis-related proteins such as sulfated glycoprotein-2 (SGP-2) and interleukin-1 converting enzyme (ICE), are activated and serve to extensively remodel the gland’s ECM and stromal components [131,140]. Integrins (collagen, laminin, and fibronectin receptors), dystroglycan (laminin-1 receptor), discoidin domain receptor 1 tyrosine kinase (collagen receptor), syndecans (co-receptors for heparin sulfate proteoglycans and fibronectin), laminin, collagen, and growth factors are all involved in the transfer of information about BM modification to MECs [52]. The mammary gland has a decreased ductal network, increased fat, and collagen-rich stroma as a result of the reduction in cell–ECM connections and the elimination of lobuloalveolar structures [141]. Adipocytes eventually replace apoptotic epithelial cells in order to preserve tissue homeostasis (Figure 6). This is an irreversible phase of involution that returns the gland to its pre-pregnant state [35,131,142].

Involution results in major structural changes in the mammary gland. It removes 50–80% of apoptotic milk secretory alveolar epithelial cells that are no longer needed [143]. In addition to macrophages, viable MECs have been proposed to engulf and remove apoptotic cells [144]. Apoptotic MECs must be removed since their existence may disrupt gland homeostasis, resulting in future breastfeeding failure and increased cancer susceptibility [145]. The presence of phagocytic leukocytes such as macrophages and neutrophils in the mammary secretions, as well as an increased concentration of immune system components such as xanthine oxidoreductase (XO), lactoperoxidase (LOP), and nitric oxide (NO), suggests that the involutory mammary gland is resistant to infections [146,147].

The dry period, also known as the static phase/non-lactating phase, occurs after involution. During the first three weeks of the dry period, dairy cows are most vulnerable to bacterial illnesses. Label-free proteome analysis of mammary gland secretions at four time intervals during dry periods, namely 0, 3, 10, and 21 days, suggested 177 DEPs. In comparison to day 0, 109 proteins were upregulated and 68 were downregulated on one or more days. The DEPs showed enrichment in various biological functions such as immunity, stress, and milk production. The downregulated proteins were linked to lactation-related activities. The study identified a number of possible protein signatures that might be further confirmed for use as biomarkers for coliform proliferation in dry secretions [148].

The loss of MECs is regained during the dry period. In comparison to the existing MECs, new MECs with higher secretory activity are produced. Between two lactations, a dry time is required to eliminate senescent cells and replace mammary stem cell progenitors, and hence MECs [149]. A 40–60-day dry interval is advantageous for subsequent lactations. In comparison, a long dry time of more than 70 days or a very short dry period of less than 20 days dramatically affects milk supply in subsequent lactation [150]. Lactation in dairy cows is reduced by 20% and lactation in goats is reduced by 12% when omitting the dry period [151].

Various signaling molecules are required for involution to occur during mammary gland development. Leukemia inhibitory factor (LIF) activated STAT3 in the bovine mammary gland after 72 h of non-milking [137]. Furthermore, overexpression of mammary gland protein-40 (MGP-40), a chitinase-like protein, causes STAT3 activation in buffalo MECs [152]. A study on the recombinant MGP-40 in buffalo revealed that 3′-untranslated region (UTR) and half-life of mRNA correspond to mRNA stability, which might be vital for mammary gland remodeling and progenitor cell protection during involution. MGP-40 is expressed in higher amounts in the mammary gland compared to other tissues in the body. Interestingly, the recombinant MGP-40 was observed to have strong chitin binding affinity but no chitinase activity, and the mutant form also did not restore the chitinase activity. This study provided insight into the catalytic inactivity of MGP-40 expressed during mammary gland involution [153]. The production of large cathepsin-D-positive cytosolic vacuoles containing triglycerides, milk proteins, and damaged membranes is regulated by active STAT3. Later, the glycerides are degraded into fatty acids such as oleic acid, which aids lysosome fusion with phagocytic MECs, resulting in cathepsin-D leaking into their cytoplasm and epithelial cell death [154,155]. TGF-3 is also involved in the MECs’ programmed cell death during the second phase of involution [156]. Aside from its role in MEC apoptosis, active STAT3 upregulates the expression of suppressor of cytokine signaling-3 (SOCS3), which inhibits the activity of STAT5 and thus the prolactin-induced activation of milk protein genes [137]. The comprehensive review study by Hughes and Watson (2018) [157] revealed the multifaceted role of STAT3 in mammary gland involution as well as breast cancer in humans. STAT3 activation is crucial during mammary gland involution as it is involved in the regulation of acute phase response during the first phase and contributes towards the shaping of the pro-tumorigenic wound healing signature of the gland during the second phase of involution.

## 4. Conclusions

The functional capabilities of the mammary gland as a milk-producing gland are well correlated with its physical traits at different stages of development. In human and mouse models, there has been significant progress in understanding the formation of the mammary gland. Cows and buffaloes are among the most important domesticated livestock species in the dairy sector. The structure of the buffalo’s mammary glands and the composition of its milk differ from those of the cow. We conducted a comprehensive evaluation of research findings on buffalo mammary gland development because there are few studies accessible in bovine mammary gland biology. Understanding mammary gland biology in dairy animals is essential for increasing milk production without compromising the animals’ health and wellbeing. In addition, the comparative research will aid in the understanding of differences in mammary gland biology and will provide answers to basic issues about mammary carcinogenesis. The current analysis will serve as a platform allowing researchers to compare the physiology of the cow mammary gland to that of other animals, and further research work will explore the hidden aspects of mammary gland development and methods for enhanced milk yield with the safeguarding of the gland from various diseases.
